# Biophysical Interactions Control the Size and Abundance of Large Phytoplankton Chains at the Ushant Tidal Front

**DOI:** 10.1371/journal.pone.0090507

**Published:** 2014-02-28

**Authors:** José M. Landeira, Bruno Ferron, Michel Lunven, Pascal Morin, Louis Marié, Marc Sourisseau

**Affiliations:** 1 Département Dynamiques de l’Environnement Côtier/Pelagos, IFREMER/Centre de Brest, Plouzané, France; 2 Laboratoire de Physique des Océans, UMR CNRS/IFREMER/IRD/UBO 6523, IFREMER/Centre de Brest, Plouzané, France; 3 Adaptation et Diversité en Milieu Marin, UMR CNRS/UPMC 7144, Station Biologique de Roscoff, Roscoff, France; CSIR- National institute of oceanography, India

## Abstract

Phytoplankton blooms are usually dominated by chain-forming diatom species that can alter food pathways from primary producers to predators by reducing the interactions between intermediate trophic levels. The food-web modifications are determined by the length of the chains; however, the estimation is biased because traditional sampling strategies damage the chains and, therefore, change the phytoplankton size structure. Sedimentological studies around oceanic fronts have shown high concentrations of giant diatom mats (>1 cm in length), suggesting that the size of diatom chains is underestimated in the pelagic realm. Here, we investigate the variability in size and abundance of phytoplankton chains at the Ushant tidal front (NW France) using the Video Fluorescence Analyzer (VFA), a novel and non-invasive system. CTD and Scanfish profiling characterized a strong temperature and chlorophyll front, separating mixed coastal waters from the oceanic-stratified domain. In order to elucidate spring-neap variations in the front, vertical microstructure profiler was used to estimate the turbulence and vertical nitrate flux. Key findings were: (1) the VFA system recorded large diatom chains up to 10.7 mm in length; (2) chains were mainly distributed in the frontal region, with maximum values above the pycnocline in coincidence with the maximum chlorophyll; (3) the diapycnal fluxes of nitrate enabled the maintenance of the bloom in the frontal area throughout the spring-neap tidal cycle; (4) from spring to neap tide the chains length was significantly reduced; (5) during neap tide, the less intense vertical diffusion of nutrients, as well as the lower turbulence around the chains, intensified nutrient-depleted conditions and, thus, very large chains became disadvantageous. To explain this pattern, we suggest that size plasticity is an important ecological trait driving phytoplankton species competition. Although this plasticity behavior is well known from experiments in the laboratory, it has never been reported from observations in the field.

## Introduction

In marine ecosystems, food web dynamics and the carbon cycle are largely controlled by the size structure of the phytoplankton community [1], [2], [3]. In a seminal paper, Margalef [4] related turbulence and nutrients as key factors determining the size and morphology of phytoplankters. In oceanic waters, the oligotrophic and low-mixing conditions favor the dominance of small cells, whereas large cells and colonies characterize the high-nutrient and high-mixing environments, like coastal upwelling systems. Diatoms range in size, but constitute the largest phytoplankton size fraction. Chain-forming species of diatom in particular display broad size plasticity, occurring solitarily or in colonies of extensive lengths [5], [6], [7]. Also, they have the ability to grow rapidly under elevated nitrate and silicate concentrations, forming blooms [8], [9]. During these blooms, the dominance of long chains alters the food pathways from primary producers to predators, reducing the interactions between intermediate trophic levels. The food web then becomes shorter and more efficient in transferring organic matter [3]. Chain forming diatoms are non-motile and generally sink faster than single cells. Although density gradients and the presence of spines or cell projections can slow gravitational settling [10], [11], [12], their fate is to passively sink out of the euphotic zone, whilst exporting to the bottom fixed CO_2_ [2].

Nowadays, the study of size spectra is a common ataxanomic tool for analyzing the structure of pelagic ecosystems and their ecological dynamics [13], [14]. However, the largest size classes included in recent analyses of phytoplanktonic size-abundance spectra are never larger than 100 µm [15], [16], whereas the diatom chains can reach several centimeters in length [17]. Studies of ancient sediments around oceanic frontal zones have revealed the presence of extensive and periodic deposits of large and colonial diatoms, suggesting that conventional oceanographic survey techniques often miss these diatom bloom events [18]. In fact, standard sampling and preservation protocols notably damage the sample by breaking phytoplankton chains [6], [17], [19]. Therefore, this methodological bias not only tends to underestimate the mean size of such large chains, but also increases the average relative phytoplankton concentration and cell encounter rate for grazers [20].

During the last decades, the development of plankton imaging systems have revolutionized the study of the plankton community, providing rapid information of the distribution, abundance, and behavior of plankton on scales that cannot be approached by conventional sampling systems [21]. The new image generation system is based on the direct observation of fluorescence in a water plane lit by a planar laser, such as the FIDO-φ [17], the Video Fluorescence Analyzer (VFA) [20], or new holographic imaging [22]. Non-holographic imaging, which was used in this study, is based on the use of a CCD camera system to record the backscattered fluorescence emitted by phytoplankton, simultaneously with CTD sensors. One of the major advantages of these imaging systems is their ability to collect in situ information without physically contacting the fragile phytoplankton that would otherwise be damaged [17], [20]. In fact, these authors have shown how these imaging systems enable a proper study of very large phytoplankton chains, unobserved in the field until that time. Further, the results suggest that long chains of diatoms must be locally common in the ocean and more important than previously thought for the functioning of the pelagic ecosystem [17], [20], [23], as Kemp et al. [18] had deduced from sediment studies.

In the present study, using the above-mentioned in situ VFA, our aim was to study, for the first time, the community of phytoplankton chains and their dynamics in relation to a physical process. The oceanographic process chosen here was a tidal front, which is probably one of the most widely distributed mesoscale oceanographic features in coastal waters. The Ushant tidal front is a prominent seasonal feature of the Iroise Sea, which is located in the northern end of the Bay of Biscay shelf [24], [25], [26] and is a predictable area for high concentrations of large diatoms [8], [18]. Tidal fronts occur in shallow areas where intense turbulence caused by tidal currents is sufficiently strong to overcome the barrier of thermal stratified waters [27], [28]. The Ushant front occurs during the warmer season, from May to the end of October, and separates vertically mixed waters at the inshore region from warmer and stratified offshore waters [26]. During this period, physical dynamics driven by wind events and tidal intensity may enhance local nutrient fluxes into the frontal region, leading to long-lived phytoplankton blooms [8], [29], [30]. This sustains the high biological activity associated with the front. For example, it is well documented that enhanced levels of phytoplankton biomass increase zooplankton productivity [10], [25], [31] and the forage success of fish, birds, and whales around fronts [32], [33], [34].

We performed the first assessment of temporal and spatial variability in the abundance and size structure of large phytoplankton chains, associated with physical/chemical dynamics during a spring-neap tidal cycle. To achieve all these goals, we performed complementary analyses of nutrient diffusion and turbulent dissipation using data from a vertical microstructure profiler.

## Materials and Methods

### Sampling Procedure

**Figure 1:**
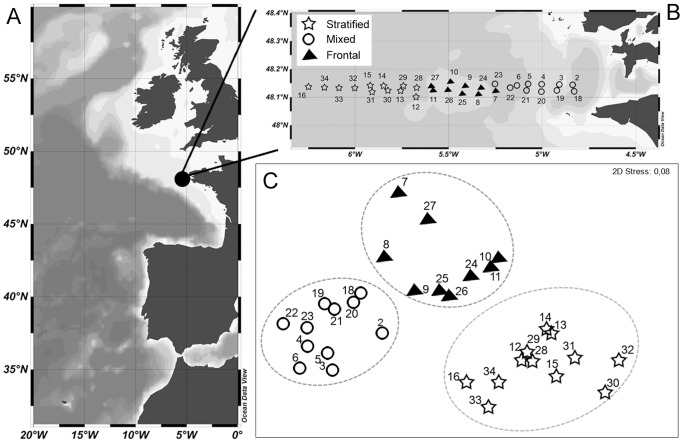
Study location and delimitation of the oceanographic regions. Map of the study area off NE Atlantic region (A). Station position and hydrological typology at the Ushant tidal front: mixed, frontal, and stratified (B). nMDS plot based on hydrological data with superimposed cluster analysis at a Euclidean distance of 4.7 (–)(C). Each dotted circle represents a significant cluster (SIMPROF *P*<0.05).

The study was carried out during the FroMVar cruise on board IFREMER R/V “Thalia” in an area off the Iroise Sea, North Bay of Biscay, from 19 to 29 September 2009. The sampling stations were arranged in two cross-shelf transects located along 48°08′N, with a station distance of 0.5° longitude, to characterize most of the frontal region (Fig. 1). The first transect (stations 1–16, from September 19 to 22) was occupied during spring tide when the tidal range was around 4.5–6 m, whereas the second transect (stations 17–34, from September 26 to 29) was set under neap tide conditions, with a smaller tidal amplitude of 1.5–3 m [31]. No specific permissions were required for sampling in the area and the field studies did not involve endangered or protected species.

### Scanfish Profiling

Temperature, salinity, fluorescence, and turbidity were sampled during spring and neap transects with a towed instrument platform, Scanfish, equipped with a Seabird SBE49 CTD, and a Seapoint FLNTU fluorometer. The Scanfish undulated between 3 m and 90 m (or 5 m above the bottom depth if bottom depth was below 90 m) while it was towed along the transect at a speed of 8 knots during the night. At each station, vertical profiles were performed during daylight conditions to collect physical, chemical, and biological data. A pelagic profiler was equipped with a SBE25 CTD probe, a Seapoint fluorometer, an in situ VFA [20], and a SBE32 carousel water sampler.

### CTD Profiles and Water Sampling, Phytoplankton Counts and Nutrients

Water samples were collected with 2 L Niskin bottles to determine the nutrients and for phytoplankton counts and identification. The downcast CTD profiles were used to decide, on board, the sampling depths during upcast. Generally, at least one sample was taken at the surface, at the chlorophyll maximum and at 80 m (or 5 m above the bottom where water column depth was <80 m). Phytoplankton samples for species counts were preserved in a Lugol-glutaraldehyde solution (1%). In the laboratory, selected samples corresponding to different physical conditions along the transect were analyzed within 2 months of preservation. The abundance (cells mL^−1^) of chain-forming species was determined by settling 10 or 50 mL of water from each sample for 48 h in sedimentation chambers. Counts were made using an inverted microscope (Leitz Fluovert). Nutrient concentrations were determined using a Bran and Luebbe AutoAnalyser II according to classical methods [35] and following the procedures described by Treguer and Le Corre [36].

### Video and Fluorescence Analysis

**Figure 2:**
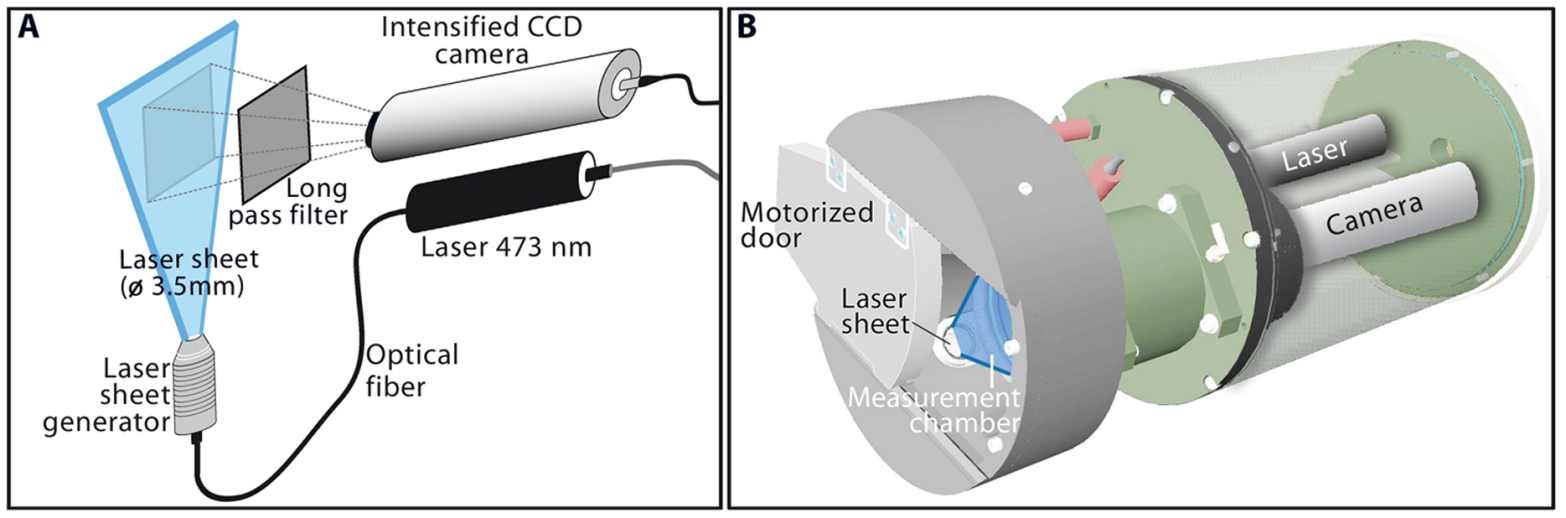
Schematic representation of the Video Fluorescence Analyzer (VFA). Imaging system components (A), and cartoon of the mechanical design of the VFA (B).

Sampling with Niskin bottles was conducted simultaneously with the VFA recording to allow a synoptic study. The VFA is an in situ planar laser imaging fluorometer system that images individual fluorescent particles and is recommended as a non-intrusive tool for a more accurate estimation of the abundance and size of the phytoplankton chains [20] (Fig. 2). The VFA is based on a 473 nm laser diode that emits a sheet of 3.5 mm depth in front of an intensified CCD detector (Fig. 2a), into an open/close dark chamber used to trap the suspended particles at selected depths in the water column (Fig. 2b). Inside the chamber, the illuminated water plane (576×768 pixels) is imaged at a rate of 25 images s^−1^ during 2 minutes by a standard CCD camera coupled to a two-stage, multichannel plate image intensifier. Using a long pass filter (>580 nm), the CCD camera imaged fluorescent particles enclosing chlorophyll pigments emitting at around 630 nm. The setting was fixed to a CCD sensitivity level of 7 and a zoom value of 150, producing a sample volume of 0.43 mL for each image and an area section of 117.7 mm^2^. Laboratory experiments with calibrated beads and monospecific phytoplankton cultures have shown that this setting allows the resolution of individual fluorescent particles from 6 µm to several millimeters [20].

**Figure 3:**
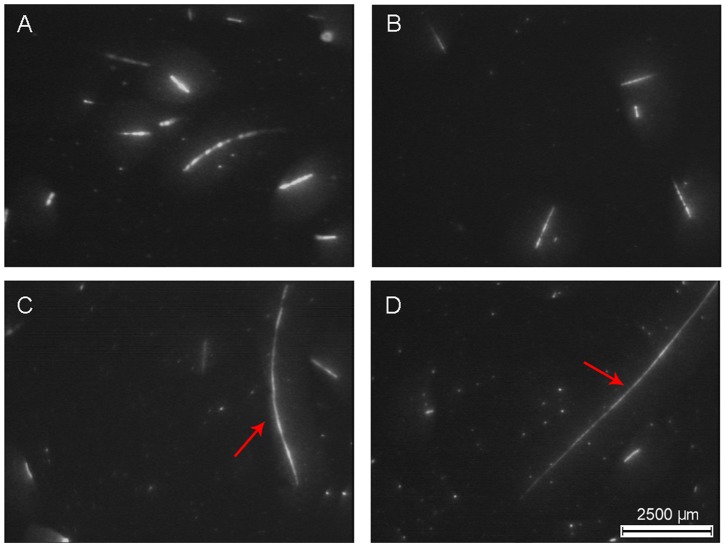
Snapshot of fluorescent particles imaged with the Video Fluorescence Analyzer (VFA). Example of images taken in the tidal front during neap (A, B) and spring tides (C, D). The most representative phytoplankton chains are shown in the panels. The shape and cell connection type of the longest phytoplankton chains (red arrows) suggest that they belong to *Pseudonitzschia* spp. (C, D).

Before image processing (Fig. 3), the boundary of individual images (30 pixels) was removed to reduce vignetting and chromatic aberration. The long phytoplankton chains appeared in the image as discontinued fluorescent regions. Due to the illumination gaps, they were usually identified as independent particles after conventional processing procedures. Following Lunven et al. [35], the combination of edge detection, image segmentation, and dilatation, using the functions *strel*, *imdilate*, and *bwmorp,* allowed the reconstruction of the chains (MATLAB Image Processing Toolbox). Finally, after particle detection, using *bwlabel* with 8-pixel connectivity, their morphological features were assessed by the function *regionprops*. The phytoplankton chains were identified by selecting fluorescent particles with an eccentricity of >0.4 and a major axis length of 115 µm (the length of two-cell chains of *Pseudonitzschia australis* as measured by VFA during the calibration tests). This parameter was fixed because *Pseudonitzschia* was one of the most abundant chain-forming taxa from the samples and formed long stiff straight chains that matched up to the shape of the many large fluorescence particles observed in the VFA images (Figs. 3 c, d). Here, the terms “size” or “chain length” refer to the major axis length of individual fluorescent particles. Their three-dimensional orientation on the light sheet can promote an underestimation of the chain lengths (especially for the larger sizes) due to the 2D projection. To minimize this bias, we applied the corrections based on the simple numerical simulations proposed by Lunven et al. [20].

### Vertical Microstructure Profile

Complementarily, a tethered vertical microstructure profiler (VMP) was used within 10 min before (resp. after) the pelagic profiler was deployed (resp. retrieved). Only downcast data were used due to the position of the sensors at the front of the instrument. For each station, four casts were carried out within a time window of 20 to 30 min. Vertical profiles were usually stopped 10 to 30 m above the bottom. The instrument fell at a velocity of about 0.60 m s^−1^. The first 6 m were systematically discarded since it corresponded to the time for the 3 m long instrument to reach a stable hydrodynamic behavior. The VMP measured vertical profiles of high-frequency fluctuations of the horizontal velocity from which the dissipation of turbulent kinetic energy (TKE), ε (W kg^−1^), was computed. The vertical resolution was of the order of 1 cm due to its 512 Hz sampling rate. Dissipation was computed, removing vibrational contamination of the high-frequency shear signal [37]. Assuming isotropy, dissipation was calculated as: *ε = *7.5 *ν<u_z_^2^>*, where *ν* is the viscosity of seawater and *u_z_* is the vertical shear of the horizontal velocity *u*. Shear variance, *<u_z_^2^>*, was estimated through a spectral analysis of the shear signals. Downcasts at a given station were bin-averaged to provide a mean vertical profile with a resolution of 2 m. This mean profile was then used to compute a profile of nutrient fluxes at each station. The diapycnal diffusivity, *K_p_* (m^2^ s^−1^), was estimated following Osborn [38]: *K_p_*_ = _0.2 *ε N* ^−2^, where the buoyancy frequency *N* is measured with the SBE 3F temperature and SBE 4C conductivity fine structure VMP sensors and calculated as *N* ^2^ = *g*/*ρ*_0_ (*∂ρ*/*∂z*); where *g* is the gravitational acceleration, and *ρ*_0_ and (*∂ρ*/*∂z*) are the average density and vertical density gradient across each overturn, respectively. The smallest scales of the flow represented by the Kolmogorov length scales were diagnosed from the dissipation data as: *L_K_* = (*ν^3^*/*ε*)^1/4^.

Finally, the vertical nitrate flux (*NO*_3*flux*_) was estimated as: *NO*_3*flux*_ = *−K_p_* (∂C/∂*z*), (µmol m^−2^ s^−1^), where *C* is the nitrate concentration, and (∂C/∂*z*) is the vertical nitrate gradient [29], [30]. Nitrate concentrations were estimated from potential density based on a polynomial relationship derived by least-squares fitting of nitrate (from the analysis of the bottle samples) and potential density (from each respective CTD). Nitrate gradients were calculated from the potential density/nitrate relationship. For spring tide, *r* ^2^ = 0.95, *n = *50, whereas for neap tide, *r* ^2^ = 0.89, *n = *55. The vertical fluxes at the base of the chlorophyll maximum were calculated around the frontal area, as the average within ±0.1 kg m^−3^ of the pycnocline value [29]. This range was always within the lower chlorophyll maximum. The vertical diffusion of silicate was not estimated due to a poor linear relationship (*r* ^2^<0.5).

### Statistical Analysis

Multivariate statistical analysis was performed to identify environmental assemblages among stations in the studied transect. The hydrological typology of the water masses was based on nine variables: the sea surface temperature; the temperature gradient from surface to bottom; the thermocline depth; the sea surface salinity; the salinity gradient from surface to bottom; the halocline depth; the surface density; the density gradient from surface to bottom; and the pycnocline depth. The Euclidean distance matrix of the normalized hydrological data, a hierarchical clustering analysis (not shown) and an associated similarity profile test, and similarity profile routine (SIMPROF; *P*<0.01; 999 permutations), were used to delineate sampling stations into different groups. The latter routine, SIMPROF, is a permutation test that objectively determines whether any significant group structure exists within a set of samples [39]. After this analysis, using the same Euclidean distance matrix, a non-metric multidimensional scaling (MDS), was performed to obtain a graphical ordination of the samples [40]. The significant results of the SIMPROF test were entered into the MDS plot to assess the level of agreement between the two techniques. The significant groups of stations detected with this procedure were used as factors to test spatial differences in the abundance of chain-forming species. To evaluate these differences, a one-way analysis of similarity (ANOSIM) test was performed based on its respective matrix of Bray-Curtis similarities, generated from the square-root transformed phytoplankton abundance data to stabilize the variance [40], [41].

The abundance and size of phytoplankton chains, obtained using VFA, were analyzed by a two-way analysis of variance (ANOVA). The tidal amplitude period (spring, neap) and the different environmental regions along the transect, previously delimited using the SIMPROF routine (mixed, front, and stratified), constituted the two factors in the analysis with two levels in the first factor and three in the second. To determine significant pair-wise differences between regions, Tukey’s HSD post-hoc test was applied. The data were subjected to a logarithmic transformation to meet the assumption of homoscedasticity [42].

Principal Components Analysis (PCA) was performed to explore the most relevant environmental variables responsible for any pattern in the size and abundance of phytoplankton chains. PCA was based on the Euclidean distance similarity matrix of the log-transformed variables: temperature; salinity; density; chlorophyll; turbidity; TKE; nitrite; nitrate; phosphate; and silicate concentrations [39]. For easier visualization of the PCA results, the stations were labeled using the levels of each factor tested in the ANOVAs. Finally, for linking environmental with phytoplankton assemblages, bubble plots of the abundance and size were superimposed over the PCA ordination. Statistical analyses were carried out using the PRIMER v.6 software and the SPSS 15.0 statistical package.

## Results

### Environmental Conditions

Based on their hydrological properties, three significantly different groups of stations (*P*<0.001) were distinguished using SIMPROF, at a Euclidean distance similarity of 4.32 (Figs. 1b, c). The use of non-metric MDS on environmental variables highlighted a clear spatial structure (low stress value of 0.08). The clusters resulting from the SIMPROF test were superimposed on the MDS plot, indicating proper separation between the three groups (Figs. 1b, c): (M) contained stations, located in the well-mixed area (2–6 and 18–23); (F) grouped stations located in the front (7–11 and 24–27); and (S) was made up of stations sampled in the offshore strong stratified region (12–16 and 28–34).

**Figure 4:**
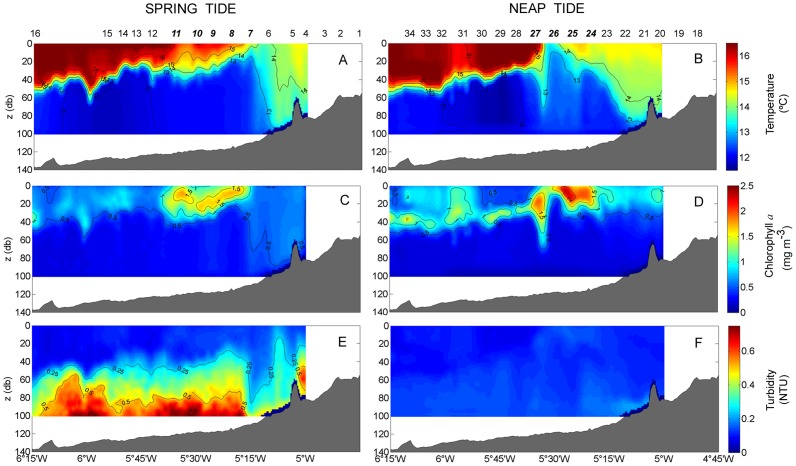
Cross-shelf sections of the water column using CTD-Scanfish. Contour plots of temperature (A, B), chlorophyll *a* (C, D), and turbidity (E, F) during spring tide (A, C, E: 21/09/2009) and neap tide (B, D, F: 28/09/2009) along the transect. Numbers along the top of the panels A and B refer to sampling stations (Fig. 1). Italic bold numbers indicate stations located in the front.

Scanfish profiles along 48°08′N transect showed a detailed view of the fine scale structure of these three regions (M, F, S) in the upper 100 m (Fig. 4). Considerable cross-shelf variation was found in all parameters: temperature; chlorophyll content; and turbidity. They showed the presence of the Ushant tidal front during spring and neap tidal periods. During our cruises, the front appeared as a region that separated the warm (>16°C SST) and stratified waters of the Celtic Sea on the western side of the transect from the well-mixed and colder waters (14.5°C SST) of the shallower Armorican region (Figs. 4 a, b). One of the noticeable differences between both tidal periods was the thermal structure of the front. Due to the position of the surface front, it is a transitional zone during spring tide, characterized by intermediate temperatures, whereas during neap tide, the front is defined as a sharp break in hydrographic properties. In fact, the bottom of the front was condensed between stations 6 and 7 during spring tide, whereas during neap tide, it tended to be more relaxed, as observed from stations 23–26.

Regarding chlorophyll, the Scanfish profiles showed a classically patchy distribution across the shelf (Figs. 4c, d). Higher concentrations remained trapped in the sub-surface layers above the bottom of the thermocline (13°C isotherm). Distinctly, the chlorophyll peaked at a thermocline depth varying from 0.8–3.9 mg m^−3^, with maximum values at the F. In this region, the chlorophyll patch showed values slightly higher in the neap tide transect (2.52±0.35 mg m^−3^ at the maximum chlorophyll) than the values obtained during spring tide (1.98±0.23 mg m^−3^ at the maximum chlorophyll). The increase in chlorophyll concentration was a trend throughout the transect, from spring to neap tide.

**Figure 5:**
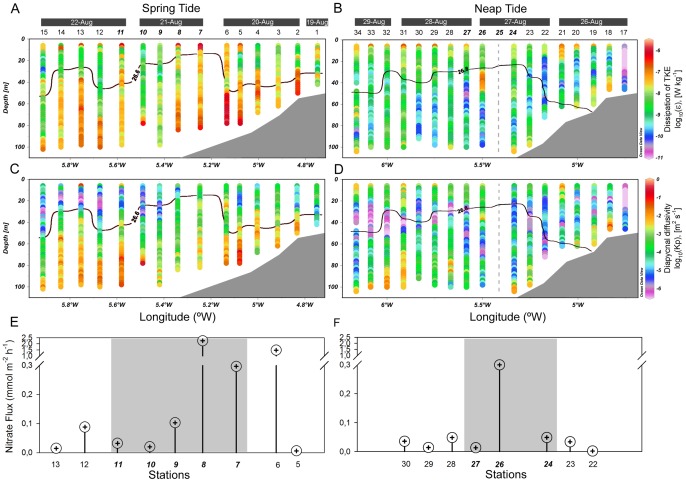
Turbulence, diapycnal diffusivity, and nutrient fluxes in the study area. Distribution of the turbulent kinetic energy dissipation rate (A, B), diapycnal eddy diffusivity (C, D), and vertical nitrate flux (E, F) along the transect during spring tide (A, C, E) and neap tide (B, D, F). The vertical dashed grey line shows the station 25 (unsumpled). The pycnocline is marked with a thick black line and corresponds to the σ_t_ = 26.6 and σ_t_ = 26.8 isopycnals during spring and neap tides, respectively. Numbers along the top of the panels A–D refer to sampling stations (Fig. 1). Italic bold numbers indicate stations located in the front. The turbulent kinetic energy dissipation rate and diapycnal eddy diffusivity are plotted on a base 10 log scale.

The TKE dissipation rate, *ε* (W kg^−1^), and the diapycnal eddy diffusivity, *K_p_*, also exhibited a strong dependence on the spring-neap tidal cycle (Fig. 5). The most significant feature is that stronger turbulence occurred predominantly in the surface and bottom layers, where *ε* and *K_p_* reached values close to 10^−6^ W kg^−1^ (Fig. 5a) and 1^−2^ m^2^ s^−1^ (Fig. 5c) respectively during spring tide. During this tidal period, the bottom layer occupied a wide region, up to 50 m above the seabed, whereas the surface layer was restricted to the upper 15–20 m depth shoreward of station 15. This was observed even as *ε* remained relatively high (10^−7^ W kg^−1^) at some locations (Fig. 5b). From spring to neap tides, the vertical extent of enhanced dissipation regions tended to decrease both near the surface and the bottom. The average dissipation in those regions also clearly weakened.

The more turbulent spring tide gave rise to large sediment resuspension from the seabed and contributed to the high turbidity conditions observed. Fig. 4e shows how the suspended particles and colloids constituted a region of turbid water that spread from the seabed (with maximum values of 0.7 NTU) up to the approximate depth of the thermocline bottom (13°C) (with lower values of 0.15 NTU). In contrast, the weaker tidal flow during the second leg prevented the upward advection of sediments, reflected by less turbid values (<0.18 NTU) in the bottom layer, allowing their deposition (Fig. 4f).

**Figure 6:**
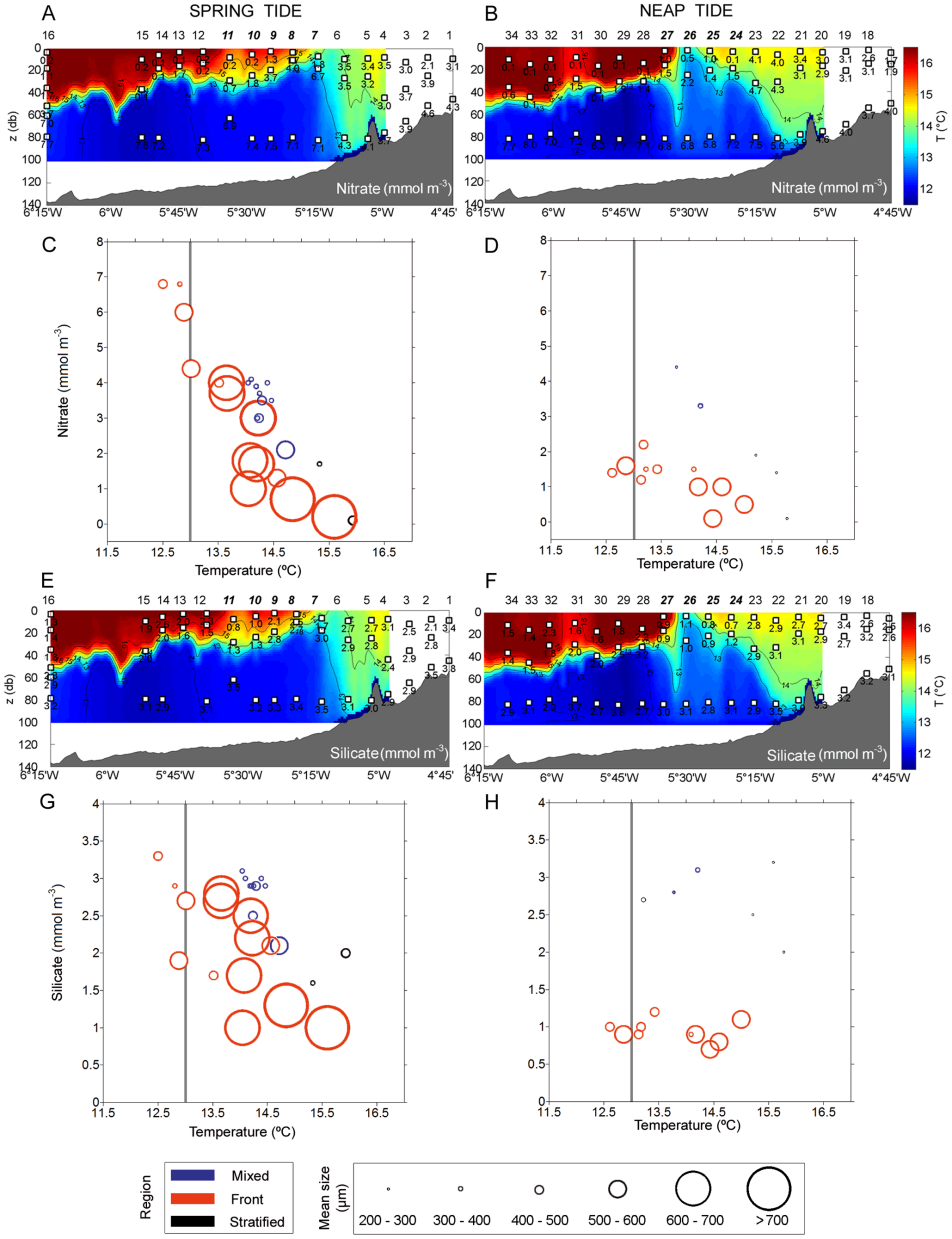
Nutrients in the study area. Nitrate (A, B) and silicate concentrations along the transects (E, F). Mean size of the phytoplankton chains relative to depth and nitrate concentrations (C) and silicate (D). The panels located in the left and right refer to observations from spring and neap tide, respectively. Numbers along the top of the panels A, B, E, and F refer to sampling stations (Fig. 1). Italic bold numbers indicate stations located in the front. In panels C, D, G, and H, the mean size of chains at each sample are shown as proportional bubbles. Note that the stations with abundance lower than 5×10^3^ cells L^−1^ were not added to the bubble plots. Vertical grey line = 13°C bottom thermocline.

Associated with the above-mentioned physical gradients and tidal strengths, concentrations of macronutrients followed the mentioned trend of turbidity. The highest concentrations were located in the mixed region, with average values of 3.72±0.93 mmol m^−3^, 0.24±0.03 mmol m^−3^, 2.9±0.29 mmol m^−3^, and 0.34±0.09 mmol m^−3^ of nitrate, nitrite, silicate, and phosphate, respectively. Phytoplankton growth appeared light-limited due to the turbidity and the width of the mixing layer. The stratified and frontal region showed the classic summer pattern, with highest nutrient levels located in the cold bottom waters below the thermocline, which prevented large nutrient diffusion to upper layers. Most of the nutrients are exhausted in the euphotic-shallow waters: <0.2 mmol m^−3^ of nitrate (Figs. 6a, b), <0.1 mmol m^−3^ of silicate (Figs. 6e, f), and <0.05 mmol m^−3^ of phosphate and nitrite. The higher turbulence and mixing allowed a significant upward transport of the nutrients through the pycnocline, mostly in the frontal region.

In the interior of the water column, between the two highly turbulent layers (the surface and the bottom), *ε* dropped significantly, by up to 10^−9^–10^−10 ^W kg^−1^ (Figs. 5a, b). During both sampling periods, turbulence near the pycnocline region tended to be intermittent, but with lower average vertical eddy diffusivities in the S (stratified) region (2.80×10^−6^ m^2^ s^−1^) than in the F (frontal) region (1.04×10^−4^ m^2^ s^−1^). In the F region, the estimation of the vertical nutrient flux across the pycnocline and into the base of the maximum chlorophyll showed that the supply rate of nitrate was higher during spring tide (0.75 mmol m^−2^ h^−1^) compared with the neap tide average (0.28 mmol m^−2^ h^−1^). Moreover, the diapycnal nitrate fluxes were spatially variable (0.02–3.24 mmol m^−2^ h^−1^ during spring and 0.04–0.60 mmol m^−2^ h^−1^ during neap tide) (Figs. 5e, f). The nitrate flux peaks were driven by high vertical eddy diffusivity values close to the pycnocline (6.18×10^−4^ m^2^ s^−1^ at station 8, and 1.12×10^−4^ m^2^ s^−1^ at station 26) (Figs. 5c, d). In the frontal area, the relatively high nutrients in the surface layers were, however, also related to the shift of the surface front and horizontal advection of coastal waters.

### Abundance and Distribution of Phytoplankton Chains

The composition of chain-forming species was dominated by diatoms. In total, seven taxa were identified and enumerated during sample processing under a microscope (Table 1). The most abundant taxa were *Pseudonitzschia* spp., *Guinardia* spp., and *Leptocylindrus* spp., which constituted more than 70% of the total abundance of chain-forming taxa or very long single cells. The distribution showed strong cross-shelf abundance variability, with higher mean values (80.0±90.0×10^3^ cells L^−1^) in the F region and lower values (14.7±27.2×10^3^ cells L^−1^) in the open ocean waters of the S area (Table 1). This spatial variability was also detected by multivariate analysis. In this sense, the one-way ANOSIM test showed that micro-phytoplankton assemblages differed between regions (Global R = 0.49, *P* = 0.001). Moreover, pair-wise comparison tests detected that the S region was also rather different in phytoplankton abundance and composition from M (R = 0.31, *P* = 0.021) and F (R = 0.52, *P* = 0.003). However, micro-phytoplankton assemblages were not significantly different between the M and F regions (R = 0.08, *P* = 0.15).

**Table 1:** Abundance of phytoplankton.

	Mixed	Frontal	Stratified
*Pseudonitzschia* spp.	15.4±17.4	60.0±49.3	7.2±6.8
*Guinardia* spp.	16.1±16.6	13.6±14.8	2.6±3.6
*Leptocylindrus* spp.	7.7±9.6	10.2±7.7	3.3±4.8
*Thalassiosira* spp.	2.7±3.3	1.4±2.1	1.5±2.3
*Chaetoceros* spp.	0.9±0.9	2.1±2.7	–
*Rhizosolenia* spp.	0.3±0.4	0.7±1.1	0.1±0.1
*Skeletonema* spp.	0.1±0.4	–	–
Total	43.4±39.6	80.0±90.0	14.7±27.2

The table shows the average abundance values (10^3^×cells L^−1^± standard deviation) of chain-forming taxa in three delimited regions in the FroMVar transect.

### Video Fluorescence Analysis of Phytoplankton Chains

**Figure 7:**
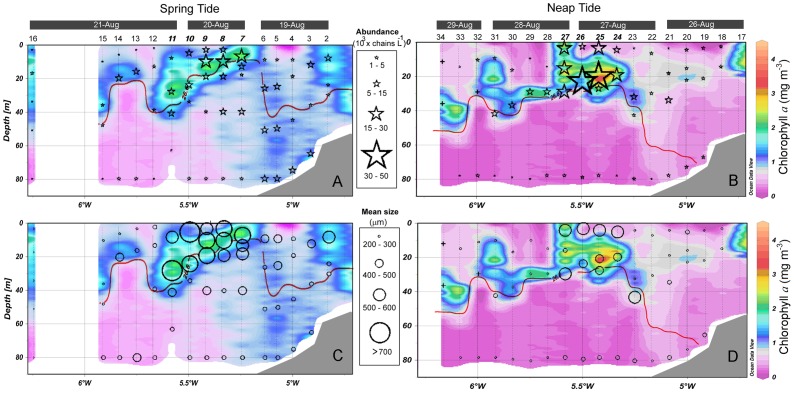
Distribution of phytoplankton chains in the study using the Video Fluorescence Analyzer (VFA). Abundance (A, B) and mean size (C, D) of chains. The panels located in the left and right refer to observation from spring and neap tides, respectively. The pycnocline is marked with a thick red line and corresponds to the σ_t_ = 26.6 and σ_t_ = 26.8 isopycnals during spring and neap tides, respectively. Numbers along the top of the panels A and B refer to sampling stations (Fig. 1). Italic bold numbers indicate stations located in the front.

The analysis of the video images showed a wide spatial variability of phytoplankton chains throughout the transect (Figs. 6, 7). ANOVA tests showed that the abundance differed significantly between hydrographical regions but no differences were found among tidal phases (Table 2). In general, large fluorescent particles were notably more abundant in the frontal area, and were almost excluded from offshore waters. Likewise, mixed waters also displayed remarkable densities but these were lower than in the front (Figs. 7a, b). Although the ANOVA test detected some differences between spring and neap tides, a clear distribution pattern was observed. During spring tide, the chains were more homogeneously spread along the M (6.2±3.2×10^3^ cells L^−1^) and F (9.0±6.5×10^3^ cells L^−1^) (Fig. 7a), whereas during neap tide, the chains were more concentrated in F, with an unexpected increase in abundance (17.0±13.9×10^3^ cells L^−1^) (Fig. 7b). ANOVA results (Table 1) also revealed a highly significant interaction effect for tide and region, which was a consequence of this pattern.

**Table 2:** Two-way ANOVA test.

		Abundance of chains			Size of chains		
	*df*	MS	*F*	*P*	MS	*F*	*p*
Tide	1	7.46E+07	2.113	0.149	1.32E+05	18.708	*
Region	2	1.27E+09	75.104	*	4.04E+05	57.131	*
Tide XRegion	2	3.11E+08	13.842	*	7.62E+03	1.079	0.344
Error	109	3.53E+07			7.08E+03		
Total	115						

Results of the effect of tide (spring and neap) and region (mixed, frontal, and stratified) on the size and abundance of phytoplankton chains. Asterisks indicate significant effects (*P<0.001).

**Figure 8:**
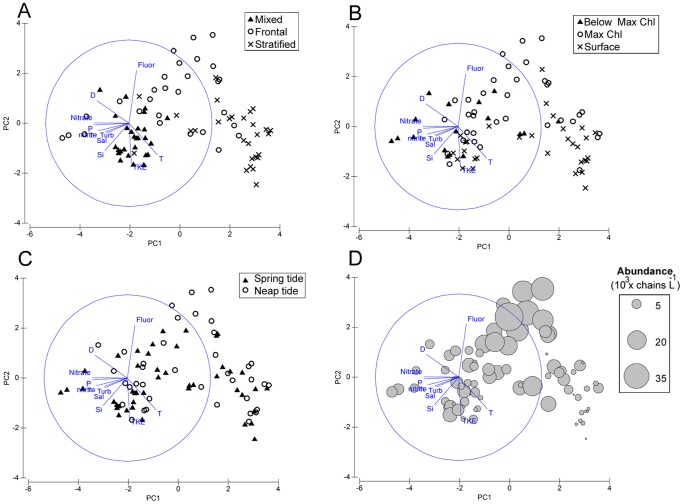
Multivariate statistical analysis. Principal components analysis (PCA) for all environmental variables labeling different factors: region (A), vertical position (B), and tidal phase (C). In panel D, the average abundance of phytoplankton chains of each sample is superimposed as proportional bubbles over the PCA. Fluor, chlorophyll; D, density; P, phosphate; Turb, turbidity; Sal, salinity; Si, silicate; TKE, dissipation rate; T, temperature.

As shown in Figure 8, the PCA corroborated the patterns detected by ANOVA tests and graphical analysis (Table 2 and Figs. 7a, b), and further highlighted some links with environmental variables and vertical distribution. The PCA ordination allowed us to reduce the environmental variability to two principal components (Fig. 8), which explained 64.5% of the cumulative variation. In the PC2 axis, higher abundances of chains were located more frequently on the opposite side of the TKE dissipation rate eigenvector where the fluorescence variable showed its highest values. As shown in Figure 7, the chains were mainly concentrated along the deep chlorophyll maximum above the 26.6 kg m^−3^ (spring tide) and 26.8 kg m^−3^ (neap tide) isopycnals, and surface waters of the front. For the PC1 axis, the chains were located in less turbid waters and nutrient-poor samples due to nutrient assimilation by the higher amount of phytoplankton.

Regarding the size structure of chain-forming species, ANOVA tests detected significant differences for tide and region factors (Table 2). The image analyses showed a wide size spectrum (major axis length), in which the chains ranged from 143 µm to particles as long as 10.7 mm, which almost occupied the entire camera field of view. In general, size followed the same spatial distribution trend as shown by abundance. In this sense, the longer average size was found in F and around the maximum chlorophyll depth (573.2±121.0 µm), where the chains also displayed higher abundances (Figs. 7c, d). The post-hoc tests corroborated the differences (Tukey’s HSD, *p*<0.001) in the size structure and abundance of the chain-forming diatoms between F and the other two regions. Nevertheless, compared with the abundance pattern, the size structure of the chain-forming diatoms displayed the opposite tendency from spring to neap tide. The mean length of the chains was notably shorter during neap tide along the entire transect, especially in the F region where these differences were particularly high (Fig. 7d). Around this region, the differences between tidal periods were even more evident at the surface (from 588.0±91.2 µm to 274.2±58.7 µm) and deep chlorophyll maximum (from 659.5±71.7 µm to 253.1±8.2 µm), where the mean size dropped by more than half of its spring levels (Figs. 7c, d).

Figure 6 (c, d, g, h) presents the interaction between temperature (as a proxy for spatial variability) and key nutrients (nitrate and silicate) in controlling the size structure of the chain-forming diatom community. Chains longer than 700 µm were confined to temperatures higher than 13°C (above the bottom of the thermocline) and relatively high nutrient concentrations for this time of year (2.2±1.1 mmol m^−3^ of nitrate; 1.82±0.71 mmol m^−3^ of silicate) in spring tide. However, as observed previously, these large size classes disappeared completely during neap tide due to significantly (one-way ANOVA: F = 5.81, *P* = 0.025 for nitrate; F = 12.89, *P* = 0.002) lower nutrient levels in that region (1.1±0.6 mmol m^−3^ of nitrate; 0.94±0.15 mmol m^−3^ of silicate).

**Figure 9:**
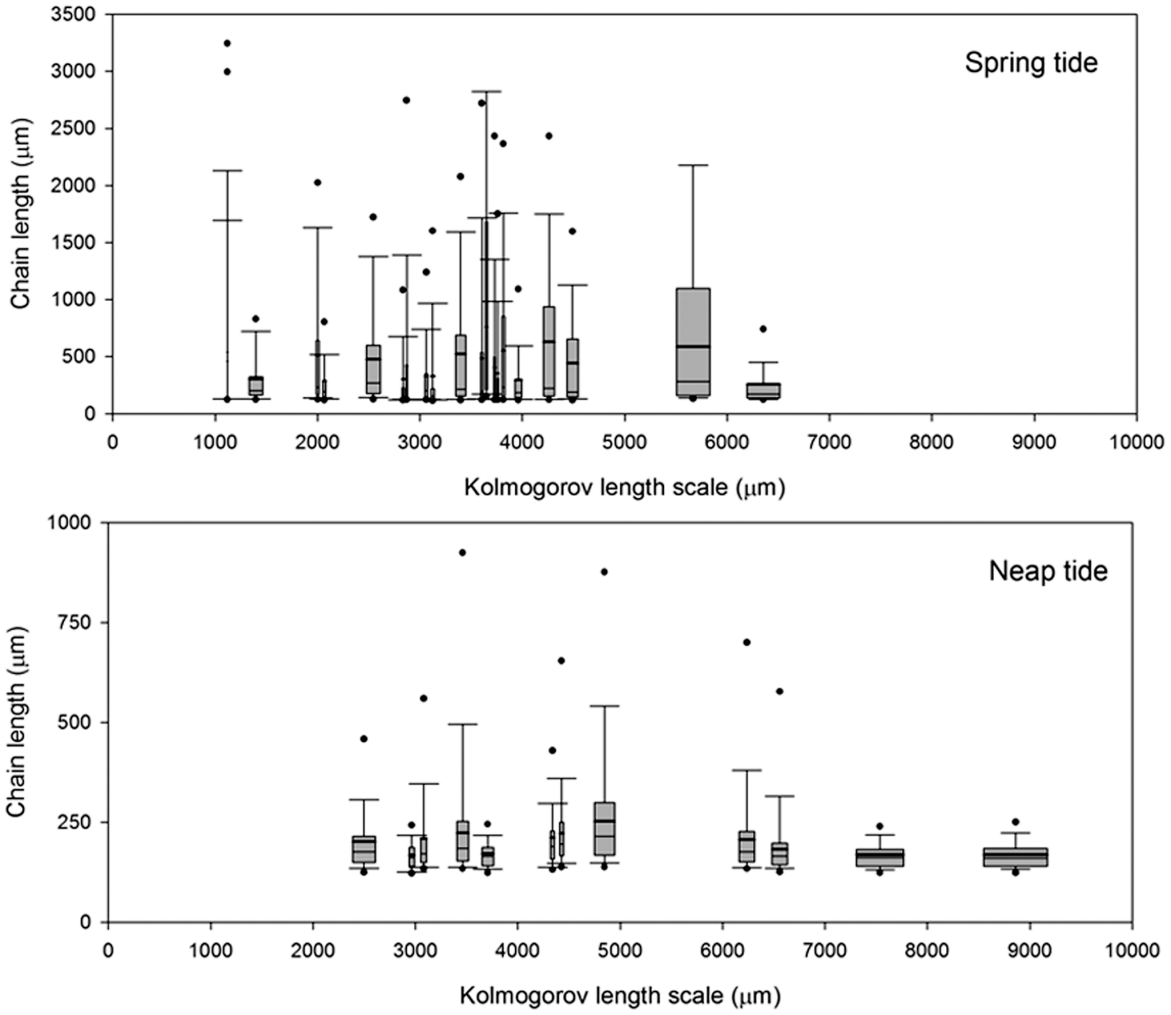
Comparison of the phytoplankton chain length spectra with Kolmogorov length scale in the frontal area. Spring tide (A). Neap tide (B). The bottom samples were removed from this analysis. In each box plot, the median (solid line) and mean (bold line) of the maximum axis length data are indicated in the center of the box and the edges of the box are the 25^th^ and 75^th^ percentiles; the whiskers extend to the most extreme data points that were not considered to be outliers.

Likewise, a comparison of the phytoplankton chain length spectra with Kolmogorov length scale, *L*_K_, in the frontal area was performed to investigate the possible effect of the small-scale turbulence on growth and nutrient diffusion (Figs. 9a, b). The Kolmogorov length scale observed around the F area ranged from 1 to 9 mm, with distinct temporal variability due to the dependence of *L*_K_ on *ε*. In this sense, the smaller sizes were found during spring tide (3.3±1.3 mm) whereas the average Kolmogorov length notably increased during neap tide (5.0±2.0 mm). In relation to the length of the chains, the Kolmogorov scales were longer than the average phytoplankton sizes (Fig. 9). However, during spring tide, some samples tended to show chain sizes closer to the Kolmogorov scales (Fig. 9a). Only the extremely long chains (>6.5 mm) observed during the spring tide were larger than the Kolmogorov scale. The values of chain size were clearly far from the Kolmogorov scales during neap tide (Fig. 7d), due to the combination of larger Kolmogorov scales and shorter chains (Fig. 9b).

## Discussion

This study was the first attempt at having a sampling strategy that tightly coupled spatial/temporal patterns of large phytoplankton chains with tidal fronts, along spring-neap tidal cycles. On large spatial and temporal scales, tidal fronts can be considered to be stable structures, and their positions can be predicted accurately by consideration of water depth and tidal velocity [43]. However, on a smaller spatiotemporal scale, fronts are dynamic; their position alters as they meander and undergo elastic tidal deformation, such as eddies [26], [44]. The physical environment thus differs greatly over small spatial scales, especially in the boundary of the front, making it challenging to sample the frontal system adequately. Our sampling strategy, combining multidisciplinary methodologies, captured the biophysical complexity of the Ushant tidal front. It delimited three distinct environmental areas (M, F, and S) along the FroMVar transect during both tidal periods in September 2009 (Fig. 1). A preliminary analysis of the video fluorescence images at selected stations has revealed a strong influence of the cross-shelf oceanography over phytoplankton size structure [31]. Classically, the S region was dominated by pico- and nanophytoplankton (up to 80% of the community), with larger phytoplankton being almost completely absent, whereas in the M and F zones, 30 to 50% of the phytoplankton cells had an estimated ESD>5 µm. Over the F area, we found the highest contribution of large cells (>20 µm ESD), 7 to 15% of the total abundance, and evidence of significant contribution of chain-forming diatoms from microscopic observations. In the present study, the analysis was extended and focused on the largest phytoplankton size classes, represented by diatom chains and colonies.

### Distribution Patterns of Chain-forming Diatoms

The image processing of the VFA samples revealed a very high spatial variability in the abundance of phytoplankton chains. The general pattern showed that phytoplankton chains were mostly located around the F area and the M area during spring tide. Microscopy counts of phytoplankton confirmed the dominance of *Pseudonitzschia* spp., *Guinardia* spp., and *Leptocylindrus* spp., which are common chain-forming diatoms previously observed in the study area [20]. However, multivariate analysis (ANOSIM) did not detect any spatial changes in the community structure or taxonomic composition between the M and F regions. This suggests that the spatial and temporal patterns were not the consequence of distinct responses of different taxa under different environmental conditions (frequency and intensity in the nutrient inputs, turbidity, or turbulence) but of the response of the entire community. This fact is quite important because it enables us to elucidate and properly address ecological processes. The diatom bloom pattern is also similar to observations from upwelling fronts [45], geostrophic fronts [8], [30], shelf-break fronts [29], and tidal fronts [25], [28]. Therefore, the similarities between these very different front-generating mechanisms should lead to the same general community-response at frontal transitions.

The other major pattern was that large phytoplankton chains tended to be found in sub-surface levels around the pycnocline. This result is also in accordance with novel FIDO-φ observations of the vertical microscale distribution of phytoplankton [17], [23]. In comparison with FIDO-φ, the vertical sampling resolution of the VFA is limited. However, it seems clear that the presence of one layer of large phytoplankton chains, with substantial temporal and spatial coherence, was associated with the maximum chlorophyll and located in the upper part of a strong pycnocline. Prairie et al. [23] also found that peaks of large fluorescent particles occurred in coincidence with low mixing and sharp density gradients. Motility-based mechanisms were discarded to explain the formation of this layer since the phytoplankton chains predominantly consisted of non-motile diatom cells. In our case, buoyancy, variable sinking rates due to density gradients [17], [23] or orientation [11], higher local production [8], [30], and failure in zooplankton grazing [46] might have contributed to the formation of this rich chain layer.

Different mechanisms have been proposed for the fertilization of the Ushant tidal front, linked with the higher local production cited above. The first is related to the repositioning of the frontal boundaries south of Ushant Island [47], whereby nutrient-rich water is horizontally introduced from the mixed zone, becomes stratified, and leads to surface maxima of phytoplankton growth. Moreover, cross-frontal mixing associated with cyclonic eddies have been described in the Ushant tidal front [26]. The 2009 FroMVar cruise was peculiar in this respect, as it was preceded by a week-long episode of northeasterly wind, which transported nutrient-rich water from the mixed area into the surface layer of the stratified zone. Stations 8 to 10 during the spring tide leg (Fig. 4) sampled remnants of this volume of water. This process can explain the observation of high densities of phytoplankton chains and the relatively elevated nutrient concentrations encountered at these stations of the F area (Figs. 6, 7).

The second fertilization mechanism studied herein proposes that over the spring-neap tidal cycle, the euphotic zone receives nutrient inputs across the pycnocline from the deeper nutrient-rich waters [48]. This nutrient flux leads to continuous phytoplankton productivity under the oligotrophic surface conditions of summer [49]. Recently, different studies have stressed the importance of the latter mechanism in chemical and biological processes. They report the vertical diffusion of nutrients as being responsible for the increase in phytoplankton biomass in the NE shelf of New Zealand [50] and the prolongation of diatom blooms in the Iceland-Faroes Front [8], Celtic Sea shelf edge [29], and at the frontal zone in the southern California current system [30]. Our estimations of the diapycnal nutrient flux are in agreement with these studies. In this sense, vertical nitrate fluxes across the pycnocline suggest a difference of a factor of more than two between spring (13.3 mmol m^−2^ d^−1^) and neap tides (5.3 mmol m^−2^ d^−1^). The higher nitrate diffusion at spring tides resulted from intermittent pulses of strong turbulent dissipation occurring within the base of the chlorophyll maximum, as also observed by Sharples et al. [29] in the front of Celtic Sea shelf edge, relatively close to our study area. The stronger nitrate flux at the Ushant tidal front (9.3 mmol m^−2^ d^−1^ compared with 3.5 mmol m^−2^ d^−1^ off the Celtic Sea shelf edge) is a result of the more intense turbulence diffusivity (10^−3^–10^−4^ m^2^ s^−1^) located close to the pycnocline in the F area of the FroMVar transect. However, given the event-based nature of the stronger nitrate fluxes and the low temporal and spatial resolution of the data, daily flux estimation must be considered as a good proxy of the vertical nutrient flux dynamics rather than a real quantitative estimation. The diapycnal diffusive flux of nitrate thus decreases significantly during neap tide over the F area.

Despite models indicating that physical processes such as convergence and divergence of cross-frontal flows can concentrate organisms due to their swimming activity or buoyancy at fronts [51], our finding supports the hypothesis that the enhanced diatom community at the front was the result of active in situ growth in response to an increase in diapycnal nutrient fluxes, rather than the passive accumulation of biomass in a zone of physical convergence. Although, we must also point out that the distribution of chains are the result of both local cell growth and transport. In this sense, the vertical and horizontal diffusion were clearly higher during spring tides, and thus the chain abundances are not completely comparable between transects around the front. The similar abundances of chains during the neap and spring tides (shorter chains but with higher concentrations during neap tides) should be related to a lower growth due to a lower diffusion of the observed population.

### Chain Length Plasticity

One of the most interesting findings was that during neap tide, the lengths of chain-forming diatoms were much shorter than the chains of spring tide. Size classes larger than 600 µm disappeared from the video images taken during neap tide. This could suggest that diatom populations shift toward shorter chains and/or solitary cells under neap tide conditions. Nevertheless, which factors control the length of these diatom chains? In this sense, it is widely accepted that the size plasticity of phytoplankton is evolutionarily driven by selective forces present in the environment, such as temperature, grazing pressure, and the interaction between nutrient limitation and physical mixing [4], [52], [53]. This size plasticity behavior has been described in laboratory studies, but has never been reported in the field. These laboratory experiments have thus shown that different environmental cues can induce chain-length plasticity by chain breakage and/or the suppression of colony formation to increase nutrient uptake efficiency during depleted conditions [5], [6] or to reduce grazing risk [7], [54], [55]. Chain length can also be considered as the net difference between the cell division rate and the chain division associated with the aging and strength of the cell-cell connections.

The first considered process that has been identified as a key factor structuring the size of the phytoplankton community is the grazing pressure. For example, calanoid copepods prefer to graze on larger cells and phytoplankton chains than single cells [55], [56] because they are easily detected [57]. This preference can cause shifts towards a shorter size distribution due to the fragmentation of the chains during their manipulation by mouthparts. Recently, several authors have observed that grazer cues frequently induce chain length plasticity [7], [54], [57]. Bergkvist et al. [7] found that the diatom *Skeletonema marinoi* suppressed the chain formation in response to the presence of chemical cues from the mesograzer copepods *Acartia tonsa*, *Centropages hamatus*, and *Temora longirostris*, significantly reducing their foraging success. Taking these studies into account, it is logical to assume that this phenomenon could also explain (at least in part) the size variability observed in our study. Fortunately, Schultes et al. [31] studied the composition and distribution of the mesozooplankton community for the same FroMVar 2009 cruise. These authors found a food niche separation that led to the typical cross-shelf distribution patterns with costal (cladocerans and small copepods) and open water communities (doliolids, and large copepods). Furthermore, they did not observe any difference in grazer density between neap and spring tides in the F area. Therefore, the lack of temporal variability suggests that length plasticity was not a response to copepod cues or grazing pressure. New findings, obtained using the submersible digital holography system, Holosub [22], support this idea. Talapatra et al. [22] observed that zooplankton (mainly copepods) avoided many prominent layers (near the pycnocline) with elevated concentrations of the non-motile diatom *Chaetoceros socialis.* Therefore, phytoplankton patches around fronts may represent areas in which there is a failure of zooplankton grazers to contain the higher phytoplankton productivity supported by enhanced nutrient fluxes [30].

The second process considered chain length modification is nutrient availability. In the northeast Atlantic, diatoms peak during the spring bloom when silicate concentrations are close to 6–8 mmol m^−3^. In summer, during thermal stratification, silicate usually begins to limit diatom production, with concentrations of around 2 mmol m^−3^, declining to <1 mmol m^−3^ under depleted conditions [58]. In our study, the nutrient concentrations decreased substantially from spring to neap tide, with similar values. This limitation was especially remarkable for nitrate and silicate in the F region (Fig. 6). We observed that silicate was rapidly exhausted before nitrate was depleted (N:Si ratio >1), which indicates a higher production of diatoms in the F region than other regions. Despite diapycnal diffusion, nutrient limitation was continuous at the front, and appeared as the main stress for micro-phytoplankton. Due to the lack of in situ nutrient uptake measurements we must go back to theoretical approaches which suggest that the shape and mechanical properties of phytoplankton chains can exert on this matter. Pahlow et al. [59] observed that chains comprising of compact cells (parameterized as rigid spheroids) were less efficient in nutrient uptake than solitary cells. Moreover, for non-motile cells in still waters, nutrient advection is limited and the nutrient source is only supplied by diffusion. When the nutrient uptake capacity of these cells is higher than the diffusive flux, a nutrient-depleted region is created around them [10]. Relative motion of the cells with respect to the fluid (by sinking or turbulent movement of the water) generates an advective transport of nutrients to renew the depleted zone [60]. In agreement with our results, these authors observed that, under initial nutrient-rich culture conditions, the relative contribution of chain-forming diatoms (*Chaetoceros* spp. and *Pseudonitzschia* spp.) to total phytoplankton biomass and the average chain length was higher under turbulence than still treatments [60]. As flexural properties also affect the motion of chains in flow [61], Musielak et al. [62] developed a more realistic model using spheres connected by elastic linear springs as chains (*Thalassiosira* type) with different levels of flexibility. Their results went further, suggesting not only that stiff chains can consume more nutrients than single cells, but also that nutrient uptake per cell increases with increasing stiffness of the chain. This suggests that chain formation is a very competitive strategy under turbulent and nutrient rich environments, which allows diatoms to out-compete other phytoplankton groups. Measurements of diatom chain mechanical properties have demonstrated that the vulnerability to breakage by flow can be enhanced under silica and nitrate limitation. This experiment, however, did not mimic the forces experienced by diatoms in the field [63]. They also observed that under this nutrient condition, the chains became more flexible, and were therefore less efficient for nutrient uptake, as Musielak et al. have modeled [62]. The high fitness of the chains could also explain the observed species dominance in the frontal area, but no measurement of chains stiffness was done. However, small-scale turbulences that can decrease this gradient around the cells were measured and confirmed this potential advantage. Chains were more than two times longer during spring tide, and for the larger size fraction these chains were on the same scale order of the smallest coherent vortices of dissipating turbulence (the Kolmogorov scales) (Fig. 9). The interaction between the chains and the turbulence in the immediate vicinity of the cells’ surface can thus be assumed only during spring tide period. This therefore suggests that lower turbulence can intensify the deleterious effect of nutrient depletion on the phytoplankton chain as we observed during neap tide.

### Conclusions

This study shows that large diatom chains are common in marine environments and that they are adapted for growth in areas that experience nutrient pulse. This capacity is a strong ecological trait that explains these species’ success in frontal regions. Notwithstanding the lower nutrient concentration in the surface waters, typical of summer, the enhanced diapycnal fluxes of nitrate across the pycnocline enable the maintenance of the diatom bloom in the frontal area throughout the spring/neap tidal cycle. Diatoms produced long chains during the spring tide, under favorable conditions of high turbulence and less-limited nutrient conditions. During neap tide, the combined effect of nutrient depletion and less-intense turbulence make the longer chains disadvantageous, inducing the diatom population to shift toward shorter chains and/or solitary cells. These cells, with higher diffusion and advection rates, are then more capable of surviving under these stressful conditions. Therefore, it seems that turbulence dynamics around frontal areas not only determine the vertical fluxes of nutrients, but also modulate the size structure of the phytoplankton community (via size plasticity behavior) without any change in its composition. Some new in situ observations of chain length should be associated with growth rate (cells d^−1^) and cell connection solidity estimates. With a larger data set, chain length could also provide a proxy for growth rate and health at the short time scale of this community. Further, the relatively high abundance of very large diatom chains suggests that the short pathway of energy from the primary producers to predators could be more important and variable over short time scales for pelagic food web functioning than previously thought. Finally, our results also illustrate the great potential for new in situ imaging systems to study biophysical interactions and trophic transfer in plankton communities.
